# Supplementary material for: Cross-sectional association of Toxoplasma gondii exposure with BMI and diet in US adults
Source: PLoS Negl Trop Dis. 2021 Oct 1;15(10):e0009825. doi: 10.1371/journal.pntd.0009825 (PMC8513882; doi:10.1371/journal.pntd.0009825)
Supplement: Attachment — Submitted filename: responses.docx [file pntd.0009825.s001.docx]

Dear Dr. Saulais and Dr. Singer:

Thank you for the opportunity to revise our paper on the relationship between Toxoplasmosis gondii and diet-related outcomes. We believe our paper has been substantially improved by addressing your concerns as well as those of the referees. Below we address each point in turn.

*I would like to ask the authors to please address carefully the comments made by the three reviewers, and particularly discuss the points that were made regarding data limitations, selection and measurement biases, and overall methodology - and what this implies for the validity and transferability of the results.*

*The reviewers have raised some concerns about some statements implying causation when the data & methodology does not allow for causal inference. Please revise the manuscript carefully to remove any ambiguity in that regard that may lead to misleading interpretations of the implications of this research. Possible alternative explanations for the relationship observed should be acknowledged and discussed accordingly in the discussion.*

Please see below for specific responses to each of the points raised here. We would like to note two points of editorial concern:

1) In our manuscript, we have included possible alternative explanations for the relationships in the “Potential Mechanisms” subsection in Results instead of in the concluding discussion. As Reviewer 2 notes, this is not standard article structure among Intro-Methods-Results-and-Discussion papers. We made the decision to include this discussion of alternative explanations in the Results section because we present supplementary results ruling out some alternative explanations. To highlight this, we renamed the Results section “Results and Discussion” while keeping the Conclusion section. If the editorial team wishes, we could also split this discussion between the other sections.
2) We have added a substantial amount of new analyses that respond to reviewer comments, and in response to Reviewer 2 we include all of this in the main body of the text. While responding directly to reviewer concerns, the addition of this new material makes the Results and Discussion section lengthy. If the editor wishes, we could put some of this material in a supplement instead of the main text.

*Reviewer's Responses to Questions*

*Key Review Criteria Required for Acceptance? As you describe the new analyses required for acceptance, please consider the following:*

*Methods*

*-Are the objectives of the study clearly articulated with a clear testable hypothesis stated? -Is the study design appropriate to address the stated objectives? -Is the population clearly described and appropriate for the hypothesis being tested? -Is the sample size sufficient to ensure adequate power to address the hypothesis being tested? -Were correct statistical analysis used to support conclusions? -Are there concerns about ethical or regulatory requirements being met?*

***Reviewer #1***

*The manuscript attempts to study the relationships between diet and Toxoplasma gondii infections, and uses a dataset from the National Health and Nutrition Examination Survey (NHANES) to conduct their analysis. This dataset has limitations, such as:*

*- biases regarding self-reported eating habits (McKenzie et al., The American Journal of Clinical Nutrition, 2021)*

*- inability to take into account food safety and hygiene, which are relevant to Toxoplasmosis transmission (Hussain et al., Pathogens, 2017)*

*- lack of discrimination in the kind of food being consume (for instance, different meats might have different parasite loads (Belluco et al., Plos One, 2016))*

*- cat ownership data only takes into account current ownership and/or a cat that lived in the same household for the past 12 months. The serological test used in the study detects IgG, which usually last for many years after infection. Additionally, correlate cat ownership and toxoplasma positivity would further strenght the data presented in tables S7 and S8*

Thank you for pushing us to describe the limitations inherent in the self-reported NHANES data. We address your points in a few steps as follows.

First, we have added a paragraph in the Data section (pg. 5) discussing misreporting in the NHANES as well as the fact that the NHANES does not allow us to examine food safety and hygiene:

“The NHANES waves used in this study provide the only publicly accessible measures of *T. gondii* exposure linked to detailed food intake data for the U.S. population. The NHANES dietary recall data, however, have two limitations relevant to examining diet and *T. gondii* exposure. First, respondents regularly misreport food consumption in self-reported dietary recalls such as the NHANES (26-28) and misreporting is greater for obese respondents (29). We note though that the dietary recall elicitation method used in the NHANES suffers from substantially less misreporting than other methods (30) and that overall energy intake is underreported by 11 % by the method used by NHANES (29). The second limitation inherent in the NHANES data is the inability to observe food safety and hygiene practices, which may be important in *T. gondii* transmission (31).”

Second, NHANES data do include the specific foods consumed. We therefore add discussion in Results (pg. 12-13) about the types of meat consumed. The revised discussion is as follows:

“Beyond a direct or indirect impact of *T. gondii* on diet behavior, our results may be explained by factors that confound estimation of a causal relationship. There are three major sources of confounding factors: food and drink sources, cat and environmental exposure, and coinfections. First, individuals may ingest *T. gondii* through food or drink sources. If individuals with higher BMI or lower HEI are more likely to eat infected foods or prepare food improperly (i.e. not wash produce, not cook meat to appropriate temperatures), they may have greater exposure to *T. gondii*. One important food source of *T. gondii* is infected meat (3). Higher meat consumption and/or improperly cooking meat could lead to an increased risk of *T. gondii* exposure. In addition, *T. gondii* prevalence differs by type of meat. For example, pork is more likely to contain *T. gondii* than beef (45).

We used multiple methods to test the extent to which meat consumption is a confounding factor. Specifically, we evaluated the association between *T. gondii* exposure and total protein consumption across the protein consumption distribution (Figure 3). We would expect this association to be positive if *T. gondii* seropositive individuals have greater exposure to *T. gondii* through higher levels of meat consumption. Instead, this association was statistically insignificant at all ranges of the protein consumption distribution. Furthermore, our main results were robust to controlling for protein consumption (Figure 4). While total meat consumption does not substantially confound our results, the composition of meat consumed by *T. gondii* seropositive individuals may be different than seronegative individuals. In particular, *T. gondii* seropositive individuals may eat more pork and thus be exposed to *T. gondii* (Table 1). *T. gondii* exposure had no statistically significant association with the mean percent of energy coming from pork (column 1), whether the individual ate any pork (column 2), or whether pork consumption was in higher ends of the pork consumption distribution (columns 3-5). Our main results were also robust to controlling for the percent of total energy consumption coming from pork (Figure 5). Differential total protein consumption and the composition of current meat consumption thus do not explain our main results. Notably, consideration of protein consumption does not account for potential differences in the individual’s *history* of protein consumption nor the proper *preparation* of meat. If food preparation drives our findings, then we would expect higher *T. gondii* infection among females since U.S. females are more likely than males to cook (46). However, more males than females have evidence of *T. gondii* infection in our sample (13.1% of males and 12.1% of females).”

Third, we have clarified the description of the cat ownership results to highlight the fact that the information on cat ownership are not found in the same NHANES waves as *T. gondii* serostatus information. We agree that displaying correlations between cat ownership and toxoplasma positivity would substantially contribute to this study, as would controlling for cat ownership. Unfortunately, the NHANES waves that include tests for *T. gondii* exposure do not include information on cat ownership; the cat ownership data are from another NHANES wave. This was not clear from our main text as we had originally put the discussion of the cat ownership data in the Supplementary Information. To remedy this, we add information on the cat ownership sample to the Data description (pg. 6) as follows:

“A final sample we used to investigate mechanisms consists of respondents in the 2005-2006 NHANES wave. The NHANES waves with *T. gondii* data described above did not have information on cat ownership, though the 2005-2006 NHANES wave included this information. The 2005-2006 wave captured cat ownership through two questions asking respondents whether a cat lives in their house now, and whether a cat lived at their house at some point in the past 12 months. We combined these two questions and measured cat ownership to be whether an individual either has a cat in the house now or had a cat in the house in the past 12 months. We made the same sample restrictions as our main analysis (U.S.-born adults with valid dietary data), yielding a sample size of 3,545 and 1,395 low-income individuals. We note that though this sample does not have available *T. gondii* information it does provide the same outcome measures as our primary sample (BMI, total energy consumption, HEI), allowing us to measure the association between cat ownership and diet-related outcomes.”

We also added the following sentence to the end of the presentation of the cat ownership results on pg. 18-19: “Cat ownership information was not available in the NHANES waves that measure *T. gondii* exposure, so we were unable to test whether previous exposure to cats moderates the relationship between *T. gondii* exposure and diet.”

***Reviewer #2***

*Yes to all those questions.*

***Reviewer #3***

*1- BMI and WHR have high false-positive rates in reference to BFP, which cannot accurately reflect the mass of adipose tissue and leads to obesity misclassification. Pregnant women were excluded from the study, but not athletes and people with high muscle mass who had a false-positive high BMI.*

*Please explain about this factor and how to eliminate its effect on the results.*

Thank you for pointing this issue out. We now investigate the robustness of our results to taking out highly-active people, using waist-to-height ratio (WHtR) instead of BMI, and using a small NHANES subsample that had percent body fat measured using dual-energy x-ray absorptiometry. Results are very similar to our main results when we take out highly-active people and when we use WHtR. Among respondents with percent body fat measurements, *T. gondii* exposure is related to higher percent body fat at the median level of percent body fat among females. We have also added the following paragraph on pg. 24:

“Our main results found that *T. gondii* exposure was related with increased BMI at higher ranges of the BMI distribution. While BMI is commonly used in public health studies, BMI may mis-identify as obese individuals with greater muscle mass (52). We investigated the extent to which our results are influenced by this potential bias in three ways. First, we excluded individuals who report participating in vigorous recreational activities at least three days per week. Excluding these highly-active individuals, our results remain robust (Figure 9). Second, we performed our analysis on the waist-to-height ratio (WHtR) instead of BMI. The WHtR has been found to predict health risk and mortality better than BMI (Ashwell et al. 2011; Ashwell et al. 2014). Similar to our main results, *T. gondii* is associated with increased WHtR among females with higher WHtR (Figure 10). Finally, the NHANES recorded the percent body fat for 3,668 respondents out of our total sample. We replicated our analysis with this subsample using percent body fat instead of BMI (Figure 11). We do not emphasize these results because of the small sample size and because fewer respondents with higher levels of BMI have valid percent body fat measures (NHANES 2020). However, *T. gondii* exposure is related with increased percent body fat among females with median percent body fat.”

*2- The level of physical activity is one of the factors affecting the BMI. Please explain about matching the BMi results with it.*

Thank you for mentioning this point. As we mention in our response to the previous comment, we investigated the robustness of our results by removing respondents with high levels of physical activity. We therefore do not believe that the level of physical activity and resulting muscle mass is driving our results.

*--------------------*

*Results*

*-Does the analysis presented match the analysis plan? -Are the results clearly and completely presented? -Are the figures (Tables, Images) of sufficient quality for clarity?*

***Reviewer #1***

*(No Response)*

***Reviewer #2***

*Yes to all those questions.*

***Reviewer #3***

*1- Please correct numbers in tables by the following guidelines are usually applicable.*

**One decimal place*

*-Means*

*-Standard deviations*

*-Descriptive statistics based on discrete data *Two decimal places*

*-Correlation coefficients*

*-Proportions*

*-Inferential test statistics such as t values, F values, and chi-squares *Use two or three decimal places and report exact values for all p values greater than .001. For p values smaller than .001, report them as p < .001.*

Thank you for pointing out the formatting of the numbers in the tables. In response to a later comment, we have replaced most tables with figures. Due to small coefficient sizes, we standardized our reporting to four decimal places in both Tables 1 and 2. (For coefficients that required more than four decimal places, we rounded and reported only up to the first non-zero decimal place.)

*--------------------*

*Conclusions*

*-Are the conclusions supported by the data presented? -Are the limitations of analysis clearly described? -Do the authors discuss how these data can be helpful to advance our understanding of the topic under study? -Is public health relevance addressed?*

***Reviewer #1***

*Although the extent in which T. gondii affects rodent behavior is still a topic of debate (Worth et al., Advances in Parasitology, 2014), the large body of literature on the subject suggest that these changes increase the likelihood of feline predation (for instance, Vyas et al., PNAS, 2007). These behavioral changes makes sense in light of evolution, as the parasite uses rodents and felines as intermediate and definitive hosts in its life cycle, and contact between the parasite and humans occured very recently (Gazzinelli et al., Cell Host and Microbe, 2014) (Shwab et al., PNAS, 2018). The links between T. gondii and changes in human behavior are controversial (Johnson et al., mBio, 2020) (Sugden et al., Plos One, 2016) and claims linking the parasite to behavioral changes in humans need to be backed by a substantial body of data. The data presented in the article correlating T. gondii infection and bmi/hei cannot be used to imply causation, as a more plausible explanation would be that higher food consuption and/or poor quality of food increases the likelihood of T. gondii infection.*

We appreciate the reviewer’s concern about our ability to infer causation and have tried to be careful about how we discuss our results. Given the fact that latent *T. gondii* infection can present as benign cysts in the brain, we believe it is not improbable that these cysts may influence human behavior. It is less clear that any changes in human behavior would be systematic. Unfortunately, the only research design that would establish causality – randomly infecting individuals with *T. gondii* and following them over time – would also be highly unethical. The best that can be done to investigate this possibility is to carefully measure associations between *T. gondii* and discuss potential confounders. This is the approach that we take in this paper. In doing so, our study addresses some of the criticisms that Johnson and Koshy (2020) have of this literature: We use a large sample, respondents do not self-select into receiving a *T. gondii* tests, and we control for multiple factors. We also go one step further and test for the possible influence of as many confounders as we can, given our data. Confounders that we cannot test for are discussed and acknowledged. We believe that a major value of our analyses is the ways in which we decompose the NHANES data and seek to evaluate relationships from multiple routes of inquiry. We have therefore tried to write the most careful paper possible on an association within the constraints of non-experimental data. That said, we agree that our manuscript does not present an experiment demonstrating cause-effect and we simply provide a set of analyses providing support for an association.

***Reviewer #2***

*Yes to all those questions.*

***Reviewer #3***

*Researchers have noted in various articles on toxoplasmosis exposure as causing high-risk and impulsive behaviors. It should be noted that perhaps reversing high-risk, impulsive behaviors may increase the risk of developing or being exposed to toxoplasmosis in these individuals.*

*please discuss about this issue*

We agree that the underlying mechanisms for our findings are not immediately clear. Thank you for pointing out that even less-impulsive behaviors may increase the risk of *T. gondii* exposure. We add the following sentence to pg. 12 where we discuss impulsivity as a potential mechanism:

“On the other hand, less impulsive behavior may contribute to eating healthier but harder-to-prepare foods, increasing risk of *T. gondii* transmission.”

*--------------------*

*Editorial and Data Presentation Modifications? Use this section for editorial suggestions as well as relatively minor modifications of existing data that would enhance clarity. If the only modifications needed are minor and/or editorial, you may wish to recommend “Minor Revision” or “Accept”.*

***Reviewer #1***

*(No Response)*

***Reviewer #2***

*MINOR COMMENTS:*

*1.	Title: I suggest revising to “Cross-sectional association of Toxoplasma gondii exposure with BMI and diet in US adults.” (Some readers may misinterpret the current title as indicating a longitudinal study of the emergence of higher BMI or poor diet over time after T. gondii exposure.)*

This is a great suggestion; we have changed the title.

*2.	Abstract: Consider reporting 95% CIs for the statistics included in the abstract. Also, consider including more numerical results in the abstract. Finally, consider mentioning the rationale for analyzing women and men separately, and for analyzing low-income people separately, in the abstract, given that results are presented separately by those characteristics.*

Thank you for this suggestion. We have added the major empirical results with 95% CIs and the rationale for separated gender and income analysis. The revised abstract now reads:

“*Toxoplasmosis gondii* exposure has been linked to increased impulsivity and risky behaviors, which has implications for eating behavior. Impulsivity and risk tolerance is known to be related with worse diets and a higher chance of obesity. There is little known, however, about the independent link between *Toxoplasma gondii* (*T. gondii*) exposure and diet-related outcomes. Using linear and quantile regression, we estimated the relationship between *T. gondii* exposure and BMI, total energy intake (kcal), and diet quality as measured by the Health Eating Index-2015 (HEI) among 9,853 adults from the 2009-2014 National Health and Nutrition Examination Survey. Previous studies have shown different behavioral responses to *T. gondii* infection among males and females, and socioeconomic factors are also likely to be important as both *T. gondii* and poor diet are more prevalent among U.S. populations in poverty. We therefore measured the associations between *T. gondii* and diet-related outcomes separately for men and women and for respondents in poverty. Among females <200% of the federal poverty level *Toxoplasmosis gondii* exposure was associated with a higher BMI by 2.0 units (95% CI [0.22, 3.83]) at median BMI and a lower HEI by 5.05 units (95% CI [-7.87, -2.24]) at the 25^th^ percentile of HEI. Stronger associations were found at higher levels of BMI and worse diet quality among females. No associations were found among males. Through a detailed investigation of mechanisms, we were able to rule out *T. gondii* exposure from cat ownership, differing amounts of meat, and drinking water source as potential confounding factors; environmental exposure to *T. gondii* as well as changes in human behavior due to parasitic infection remain primary mechanisms.”

*3.	Introduction, paragraph 2: Consider specifying what country was the setting for reference #22 rather than referring to “not … all populations.”*

We revised the sentence (pg. 3) using this reference to say:
“However, the relationship with obesity was not found in a sample of Mexican individuals…”

*4.	Methods, statistical analysis: Why did the authors adjust for season of interview? Consider providing a rationale in the manuscript.*

We add the following sentence to pg. 7: “We controlled for the part of the year the interview was conducted (November April vs. May-October) to account for possible seasonal differences in diet behavior.”

*5.	Tables/figures: Consider listing the control variables in a footnote below each table or figure so that the reader does not need to refer back to the Methods section to see what was adjusted for.*

Thank you for the suggestion. We have added controls to the notes for each figure and table.

***Reviewer #3***

*N/A*

*--------------------*

*Summary and General Comments Use this section to provide overall comments, discuss strengths/weaknesses of the study, novelty, significance, general execution and scholarship. You may also include additional comments for the author, including concerns about dual publication, research ethics, or publication ethics. If requesting major revision, please articulate the new experiments that are needed.*

***Reviewer #1***

*The study cannot imply causation from correlation, and more robust data would be required to imply that a parasite causes changes in human behavior, specially considering that humans are accidental hosts.*

Thank you for pushing us to clarify further that our results are not causal. This limitation was our reason for including a section that explicitly discusses and tests for specific mechanisms – many of which would be confounders to a causal interpretation. In addition to being careful to describe our results using appropriate language (“relationship”, “association”), we have added language throughout the paper to emphasize that our results are not causal.

In particular, at the end of the Introduction on pg. 3 we add the following justification for examining mechanisms: “Since the available data do not allow us to establish causality, our secondary aim was to investigate potential mechanisms for any relationship between *T. gondii* exposure and these outcomes.”

We also add the following to the beginning of the subsection on potential mechanisms (pg. 11-12): “While we found that *T. gondii* exposure is associated with higher BMI and worse diet among females, our data are cross-sectional and do not allow us to make any claim of causality. Therefore, in this section we explore potential mechanisms for our findings among females. We start with potential reasons for a causal relationship and then explore mechanisms that may confound estimation of this causal relationship.”

On pg. 12 we introduce mechanisms beyond direct or indirect impacts of *T. gondii* as follows: “Beyond a direct or indirect impact of *T. gondii* on diet behavior, our results may be explained by factors that confound estimation of a causal relationship.”

On pg. 28, in the Conclusion, we summarize the results on mechanisms as follows: “We also examined potential mechanisms underlying our findings. Some of these mechanisms represent factors that confound a causal interpretation of our findings. While we were able to provide evidence that our findings were not fully explained by some mechanisms, other mechanisms remained unexplored. This represents one limitation of our study. Data availability does not allow us to examine many sources of exposure to *T. gondii*. Important potential sources that we cannot observe in our data include contamination of meat or produce and environmental exposure. Since we cannot rule out other mechanisms, we are not able to establish a causal relationship between *T. gondii* exposure and diet-related measures. This broader limitation is common in cross-sectional observational studies and provides directions for further research.”

***Reviewer #2***

*The authors studied the associations of Toxoplasma gondii exposure with BMI, total energy intake, and Healthy Eating Index (HEI) in a cross-sectional study of 9,853 American-born women and men in NHANES. They found that T. gondii exposure was associated with higher BMI in women, not in men, and with lower HEI in low-income women, not in all women or in men. Investigation of some potential biological mechanisms including protein consumption, water source, cat ownership, and Toxocara coinfection suggested that such mechanisms do not explain the observed associations.*

*In my opinion, the authors based their study on a strong rationale, they expressed their research questions clearly, their approach was methodologically strong in most respects, their reporting of results was thorough and well organized, and their conclusions were justified by the results. The manuscript was well written.*

Thank you for the positive support and evaluation, we appreciate it.

*I have a few comments and questions for the authors to consider:*

*MAJOR COMMENTS:*

*1.	Methods: Regarding the potential for selection bias, I have four questions: (1) How many people from the total 2009-2014 sample were excluded from analysis due to missing T. gondii exposure? (2) How many were excluded due to missing BMI or diet data? (3) To what degree did exclusion for missing T. gondii measure differ across BMI, total energy, or HEI values? (4) To what degree did exclusion for missing BMI or dietary data differ by T. gondii exposure? Reporting this information in the manuscript would help some readers better assess the potential for selection bias affecting the results, and could also allow the authors to comment more about that possibility in the discussion.*

Thank you for suggesting a detailed analysis of selection bias. We have added this (including answers to the reviewer’s questions 1-4) on pg. 21-22 in Results:

“This section has discussed potential mechanisms for observing a relationship between *T. gondii* exposure and diet-related outcomes. Beyond these mechanisms, the potential exists for our results to be driven by sample selection. NHANES examination, dietary, and *T. gondii* data are recorded for most, but not all, of the NHANES respondents. As noted above, only samples with surplus serum were tested for *T. gondii* antibodies. BMI data are available for respondents with valid height and weight data, and diet information (total energy consumption and HEI) is available for NHANES respondents who completed the food intake module. If diet-related outcomes among respondents with *T. gondii* data were systematically different from outcomes among respondents without *T. gondii* data, our results may be an artifact of this selection. Out of the total 13,082 respondents in our survey waves, 1,916 have missing *T. gondii* exposure information, 601 respondents have missing BMI data, 1,224 have missing energy consumption information, and 1,224 have missing HEI data. All respondents with missing energy consumption have missing HEI scores and vice versa. Table 1 tests whether *T. gondii* or our outcomes are systematically missing. We found that none of our main outcomes (BMI, energy consumption, HEI) predict whether a respondent has missing *T. gondii* data. Likewise, *T. gondii* exposure does not predict having missing BMI or diet data. These results suggest that patterns of missing values are not driving our results.”

*2.	Methods/Results: In the investigation of heterogeneity by low-income status, consider reporting results for those above 200% of the poverty line in addition to those below 200% of the poverty line, so that readers will be able to see both sides. Also, consider investigating the T. gondii by low-income interaction in a single model with an interaction term and calculating the excess association due to interaction. Such an analysis would better support the authors’ interest in the low-income population by determining the degree to which the joint effect of T. gondii plus low-income is stronger than would be expected if the two exposures didn’t interact.*

Thank you for the suggestions. We have revised our models to be a single model with an interaction term for whether the respondent is >200% of poverty. This allows us to observe the main effect for the poverty sample and also test whether the respondents >200% of poverty react differently. Standard errors for the interaction are generally large, limiting our ability to accurately test the difference between low-income and wealthier respondents.

*3.	Methods, statistical analysis: Why did the authors analyze females and males separately, with no models combining females and males? Consider providing a rationale in the manuscript. Was this decision made a priori or influenced by the data analysis?*

The decision to report males and females separately was made prior to the data analysis and was based on prior studies which show a difference over gender in how *T. gondii* is related with behaviors. To clarify this, we add the following sentences to the Statistical Analysis subsection (pg. 7): “Prior studies found differences between males and females in relationships of behavior with *T. gondii* exposure (33-34), so we estimated separate regression models for males and females. This decision was made prior to data analysis and was not impacted by the results.”

*4.	Tables: Consider reporting 95% CI for each estimate instead of standard error. Also consider showing the results for the group that is above 200% of the poverty line.*

In response to this reviewer’s point 2 (above) we used a single model with an interaction term, though do not show separate results for the group above 200% of poverty. We also looked at using confidence intervals instead of standard errors for the tables, but due to some of the small coefficients the confidence intervals added a lot of horizontal space to the tables and made them difficult to read.

*5.	Figures: Consider using the same Y-axis scales in the figures for women and for men, in order to make the visual comparison easier. Also consider showing the graphs for the group that is above 200% of the poverty line.*

We have revised Figures 1 and 2 to show the same y-axis scales for women and men. Since we use a single model with an interaction term, we do not show a separate graph for those above 200% of poverty.

*6.	Results, potential mechanisms: I found this section easy to understand and logically organized, however the organizational scheme was unusual in the context of the overall IMRaD (Intro-Methods-Results-and-Discussion) structure. A lot of sentences in this section of Results sounded more like Intro, Methods, and Discussion. Consider moving sentences into the appropriate sections of the article. However, you may want to consult with the journal’s editorial team on this.*

Thank you for the suggestion. We agree that parts of the potential mechanisms subsection are often found in other sections. However, we believe that putting each part of the discussion of the potential mechanisms in other sections would break the flow and make this discussion difficult to follow. We kept it as a separate section in this draft and will consult with the editorial team on the journal’s preferences.

*7.	Supplemental tables and text corresponding with Results, potential mechanisms: First, I recommend incorporating all supplemental text into the main body of the manuscript in appropriate places (some may fit better in Intro, Methods, or Discussion, than in Results, similar to above comment). Second, I recommend replacing all Supplemental Tables with figures similar to the figures shown in the main manuscript. I think all the points the authors want to make in this section about potential mechanisms will be made more clear with visual figures instead of lots of tables. Finally, if the authors accept my suggestion to replace the supplemental tables with figures, consider incorporating some (or all) of the new figures into the main manuscript instead of a supplement. The exploration of potential mechanisms was one of the most interesting parts of the article, and in my view deserves greater emphasis and visibility. To make room for more figures in the main manuscript, consider moving Tables 1 and 2 into a supplement. The figures really showcase the findings better than the tables, in my opinion.*

These are great suggestions. We have replaced all supplemental tables with their corresponding figures and have incorporated these figures into the main text. Since in our main results we only find associations for females, in the potential mechanisms section we only include figures for females to conserve space.

*8. Discussion: The cross-sectional study design is not the strongest for this type of research question, where the hypothesis is that T. gondii exposure LEADS TO increased BMI or deterioration in diet quality, but the data structure do not permit knowing which came first, the T. gondii exposure versus the BMI and diet quality levels. Although the authors mention the limitations of cross-sectional studies, I suggest stating more clearly and strongly the difficulty in drawing causal inference from this particular study on this particular question. To what extent is reverse-causality a possible explanation for the results?*

In response to this reviewer’s comment as well as that of another reviewer, we have included more language to clarify the limitation of our data in terms of claiming causality. In addition to being careful to describe our results using appropriate language (“relationship”, “association”), we have added language throughout the paper to emphasize that our results are not causal.

At the end of the Introduction on pg. 3 we add the following justification for examining mechanisms: “Since the available data do not allow us to establish causality, our secondary aim was to investigate potential mechanisms for any relationship between T. gondii exposure and these outcomes.”

We also add the following to the beginning of the subsection on potential mechanisms (pg. 11-12): “While we found that *T. gondii* exposure is associated with higher BMI and worse diet among females, our data are cross-sectional and do not allow us to make any claim of causality. Therefore, in this section we explore potential mechanisms for our findings among females. We start with potential reasons for a causal relationship and then explore mechanisms that may confound estimation of this causal relationship.”

On pg. 12 we introduce mechanisms beyond direct or indirect impacts of *T. gondii* as follows “Beyond a direct or indirect impact of *T. gondii* on diet behavior, our results may be explained by factors that confound estimation of a causal relationship.”

On pg. 28 in the Conclusion we summarize the results on mechanisms as follows: “We also examined potential mechanisms underlying our findings. Some of these mechanisms represent factors which confound a causal interpretation of our findings. While we were able to provide evidence that our findings were not fully explained by some mechanisms, other mechanisms remained unexplored. This represents one limitation of our study. Data availability does not allow us to examine many sources of exposure to *T. gondii*. Important potential sources that we cannot observe in our data include contamination of meat or produce and environmental exposure. Since we cannot rule out other mechanisms, we are not able to establish a causal relationship between *T. gondii* exposure and diet-related measures. This broader limitation is common in cross-sectional observational studies and provides directions for further research.”

To directly respond to the issue of reverse causality, we agree people can become exposed to *T. gondii* through food consumption. It is unclear, however, whether these same food choices are those that lead to worse diets (e.g., lower HEI). In our potential mechanisms section we show that our HEI results are robust to controlling for protein consumption and pork consumption (*T. gondii* is found more in pork products than in beef or chicken). Greater produce consumption would (by construction) increase HEI, so *T. gondii* exposure from produce would not be driving our results. While a straightforward reverse causality story therefore appears less likely, we agree that there are confounding factors related to both diet quality and *T. gondii* exposure that remain. For example, we are unable to examine whether individuals with worse diets were also exposed to more meat or pork earlier in life, or whether they also use less hygienic methods to cook food. We are also unable to examine the extent to which individuals with worse diets are exposed to *T. gondii* through environmental sources outside of food. To make this more transparent, we have included these points in our section on potential mechanisms.

*MINOR COMMENTS -- See above in the section "Editorial and Data Presentation Modifications?"*

*I prefer to sign my review – Evan Thacker, PhD, Brigham Young University*

***Reviewer #3***

*The goals and methodology of this study is noteworthy, although the authors need to address some minor problems in the methodology and reporting data as well as discussion section.*

Thank you for your points. In response to all reviewers’ comments, we greatly revised the methodology and data reporting.
